# Translational cancer biology

**DOI:** 10.1186/s12967-020-02537-z

**Published:** 2020-09-23

**Authors:** Cristina Maccalli

**Affiliations:** Sidra Medicine, Doha, Qatar

We are excited to announce the launch of a new section in the *Journal of Translational Medicine* entitled “*Translational Cancer Biology*”. This section aims to provide a platform for the communication and dissemination of advances in cancer biology and their translational applications. Studies considered for publication include those dissecting the mechanisms of transformation, progression and metastatization, the biology of cancer stem cells and their immunological properties, the mechanisms undergoing the epithelial-to mesenchymal (EMT) transition and tumor dormancy, their relationship with immune functions and the mechanisms undergoing cancer resistance to therapies. This section is also dedicated to those investigations dedicated to the translational aspects and the development of novel therapies related to the aforementioned themes and the identification of patient’s responsiveness and outcome to therapies.

Cancer is one of the leading causes of morbidity and mortality in the western world. The progress in understanding the oncogenic and pro-survival pathways as well as the immunological profile of cancer cells allowed to design novel targeted therapies and immunotherapy, leading to the improvement of the overall survival of cancer patients. Nevertheless, a significant proportion of cancer patients are unresponsive or develop resistance to therapies. Advances in genome sequencing allowed to show that tumor lesions results from heterogeneous mixture of genetically distinct subclones that arise through tumor evolution [1–3]. The unique driver mutations within each subclone can impact the cancer hallmarks differently, thereby contributing to functional heterogeneity. In addition, a variety of DNA mutations arise at different stage and dynamically along with tumor development and progression [4, 5]. These genetic variants together with epigenetic modifications drive the development of hierarchically organized neoplastic tissues comprising subpopulations of self-renewing cells with “stemness” properties that allow the long term maintenance of tumors. The evidence that rare cells within tumor lesions, cancer stem cells/cancer initiating cells (CSCs/CICs), represent a key component of tumor initiation and propagation was obtained initially in hematological malignancies [6, 7] and subsequently in solid tumors with different histological origins [8–11].

CSCs/CICs are endowed with the ability to modulate their proliferative status from quiescent to slow or fast cycling [12, 13] and with the resistance to therapeutic treatment, such as chemotherapy and radiotherapy [14–20]. These cells can survive and initiate the formation of local recurrence, and through migrating at distant site, of metastases, even many years after the initial clinical response to the treatments [19, 21–24]. One of the major factor influencing the phenotype, molecular properties and proliferative status of CSCs/CICs is represented by the “niche” or tumor microenvironment (TME) [25, 26]. The epithelial-to-mesenchymal transition (EMT) is a developmental program underlying the acquisition of mesenchymal properties by epithelial cells [27, 28]. EMT become re-activated in cancer cells, promoting cell migration, dissemination of cells and metastasis formation [29, 30] and is associated with the generation of CSCs/CICs [31, 32]. The identification of key signaling pathways underlying CSCs/CICs properties would more accurately provide insights for the clinical contribution and significance of these cells [33–36]. Epigenetic mechanisms, including DNA methylation, histone modifications, chromatin remodeling and changes in non-coding RNA, such as miRNAs, regulate the landscape of cells. The deregulation of these genomic make-up can increase stemness and self-renew, contributing to the development of CSCs/CICs [37–39]. A complex connection of molecular signaling can be deregulated by aberrant epigenetic modification during the course of tumor formation contributing to the maintenance and proliferation of CSCs/CICs, as well as resistance to therapies and tumor progression. Progress in dissecting the molecular pathways underlying CSC/CIC properties lead to the identification of some of the mechanisms that can render this subpopulation of cells resistant to therapy, such as high levels of anti-apoptotic signaling [40], DNA repair molecules [41], up-regulation of cellular extrusion pumps [42], increased aldehyde dehydrogenase (ALDH), metabolic activity [43], up-regulation of IL-4 signaling-dependent resistance to apoptosis [44]. Nevertheless, others key mechanisms underlying this phenomenon still need to be dissected and fully understood.

The mechanisms underlying dormant state of tumor cells are not fully understood. Cellular quiescence, the genomic make up of tumor cells, angiogenesis and the crosstalk with TME and immune responses can influence and shape tumor cell properties. Among these mechanisms, CSCs/CICs can be relevant component of tumor dormancy [45]. The presence of dormant cells, through their escape from immune recognition, may lead to tumor recurrence and metastatization. The understanding of the molecular pathway of cancer biology and stemness represent a major endeavor for the discovery and validation of biomarker signatures associated with the therapeutic efficacy and for the identification of more effective precision medicine interventions.

The *Journal of Translational Medicine* through providing high standard peer-review process represents a platform for efficient communication of up-to-date results and scientific discussions. The *Translational Cancer Biology* section will guarantee high quality and competitive publications. The Editorial Board is looking forward to receiving your contributions.
